# Risks for Child Cognitive Development in Rural Contexts

**DOI:** 10.3389/fpsyg.2018.02735

**Published:** 2019-01-09

**Authors:** Maria Julia Hermida, Diego Edgar Shalom, María Soledad Segretin, Andrea Paula Goldin, Marcelo Claudio Abril, Sebastián Javier Lipina, Mariano Sigman

**Affiliations:** ^1^Laboratorio de Neurociencia, Universidad Torcuato Di Tella, Buenos Aires, Argentina; ^2^Fundación Mundo Sano, Buenos Aires, Argentina; ^3^Instituto de Educación, Universidad Nacional de Hurlingham, Buenos Aires, Argentina; ^4^Consejo Nacional de Investigaciones Científicas y Técnicas, Buenos Aires, Argentina; ^5^Departamento de Física, Facultad de Ciencias Exactas y Naturales, Universidad de Buenos Aires, Buenos Aires, Argentina; ^6^Unidad de Neurobiología Aplicada, Centro de Educación Médica e Investigaciones Clínicas “Norberto Quirno”, Buenos Aires, Argentina; ^7^Facultad de Lenguas y Educación, Universidad Nebrija, Madrid, Spain

**Keywords:** socioeconomic status, rural context, urban context, child cognitive development, executive functions, preschool attendance, father’s educational level

## Abstract

While poverty all over the world is more typical and extreme in rural contexts, interventions to improve cognition in low socioeconomic status (SES) children are for the most part based on studies conducted in urban populations. This paper investigate how poverty and rural or urban settings affect child cognitive performance. Executive functions and non-verbal intelligence performance, as well as individual and environmental information was obtained from 131 5-year-old children. For the same level of SES, children in rural settings performed consistently worse than children in urban settings. These differences could be accounted mostly by the months of past preschool attendance and the father’s completed level of education. These results should inform policies and programs for children living in rural poverty worldwide, and specially in Latin America.

## Introduction

Over the last few years, substantial effort has been committed to design public policies and interventions to attenuate the negative effect of poverty in child cognitive development (Bradley and Corwyn, 2002, p. 372; Slavin, 2002, p. 15; Bierman et al., 2008, p. 1810; Yoshikawa et al., 2012, p. 272; Richter et al., 2017, p. 103). While this scientific program is in Stoke’s “Pasteur Quadrant” (Stokes, 2011, pp. 1–196; Sigman et al., 2014, p. 497) of basic research inspired by use and practical applications, there is an incongruity: although worldwide the incidence of extreme poverty is much more prominent in rural contexts, most of studies on the effects of poverty in cognitive development have been carried out in urban populations (Lichter and Johnson, 2007, p. 331; Schreuder, 2010, p. 45; Tine, 2017, pp. 9–22). For example, in Argentina the incidence of non-urban, poverty is 18.2% while the incidence of urban poverty is 8.3% (Instituto Nacional de Estadísticas y Censos, 2010, p. 217). Importantly, most of the inhabitants living in extreme poverty are children (Olinto et al., 2013, p. 125).

In fact, the effects of poverty and the effects of living in a non-urban context are often confounded in scientific studies, because low SES is most prevalent in rural samples (Foulkes et al., 2008, p. 129; Morgan, 2015, p. 1). Some studies have analyzed developmental or academic trajectories in rural low SES children, without including comparisons with urban peers (Brody et al., 2002, p. 1505; Nadel and Sagawa, 2002, pp. 1–105; Vernon-Feagans and Cox, 2013, pp. 1–23). A second group of studies (Förster and Rojas-Barahona, 2014, pp. 346–504; Mykerezi et al., 2014, p. 17; Castro and Rolleston, 2015, pp. 5–48) examined the rural-urban gap in cognition, but only atone SES level (i.e., low-SES children). A third group of studies has identified effects of poverty and effects of context, but do not separate those effects (Foulkes et al., 2008, p. 129; Foulkes and Mori, 2009; Gouin et al., 2015). A fourth group of studies, go deeper in the associations among poverty, context and cognition, examining whether the form and magnitude of income’s relationship with early reading and math achievement differ across the urban-rural continuum (Miller and Votruba-Drzal, 2013, p. 234; Miller et al., 2013, p. 1452). However, those studies were not focalized in separating the effects of poverty and context. In addition, none of those studies analyzed cognition in terms of executive functions. There is just one study that analyzed executive functions in rural low SES samples (Vernon-Feagans and Cox, 2013, pp. 1–23), but it also does not compare achievements with an urban sample. In conclusion, although some studies have investigated cognitive development in rural contexts, they were not designed to factor out the effect of poverty from the effect of living in urban or rural context in executive functions.

More generally, while the terms poverty and low SES has been used broadly to refer to scarceness and generally associated to risks of cognitive development, to design evidence based policies and interventions, it is imperative to determine what components of low SES, and their interactions, present a major risk for cognitive development (Lipina, 2016, p. 241; Duncan et al., 2017, p. 413).

A review of the existing literature suggests that several aspects of scarceness which are prevalent in rural contexts may present high risk factors for cognitive development. For example: lack of access to social services, public health, and resources (Foulkes and Mori, 2009, p. 83; Miller and Votruba-Drzal, 2013, p. 234; Gouin et al., 2015, p. 10; Morgan, 2015, p. 1; Robinson, 2017, pp. 1–11), lower quality of education, teachers with lower wages and levels of training, reduced access to preschool education (Castro and Rolleston, 2015, pp. 5–48; Gouin et al., 2015, p. 10), fewer years of parental education (Nadel and Sagawa, 2002, p. 61; Foulkes et al., 2008, p. 129; Foulkes and Mori, 2009, p. 83; Mykerezi et al., 2014, p. 17; Tine, 2017, p. 9), and significantly less familiar stimulation at home (Burchinal et al., 2008, p. 41; Miller and Votruba-Drzal, 2013, p. 234; Förster and Rojas-Barahona, 2014, p. 476). Though this picture seems grim, some studies have found a positive association between living in a rural context and academic achievement (i.e., language, verbal memory, and school adaptation) (Paxson and Schady, 2007, p. 49; Miller et al., 2013, p. 1452; Tine, 2014, p. 599), and it has been suggested that exposure to nearby nature may be a buffer for everyday life stress in children (Wells and Evans, 2003, p. 311). Hence, there are contradictory views and results regarding the impact of living in a rural context on cognitive development. A meta-analysis of these studies is very difficult because they have used different criteria to define rurality (Hart et al., 2005, p. 1149).

There is an additional challenge to compare those results: the variability in the cognitive measures used across studies. Amongst the cognitive variables often measured throughout development, Executive Functions (EF) have been established as good predictors of the child’s self-regulatory abilities, and their future academic performance and well-being (McClelland, 1973, pp. 1–14; Brooks-Gunn and Duncan, 1997, p. 55; Blair and Diamond, 2008, p. 899; Moffitt et al., 2011, p. 2693). However, research in rural contexts have mostly focused in other variables such as non-verbal intelligence (Berry et al., 2016, p. 115).

The central aim of this study is to compare cognitive achievement in selected samples from different SES and contexts to understand which aspects of rural poverty pose the main risks to cognitive development. The overarching goal of our research program is to contribute showing critical information to the design of public policies and education programs that can be tailored to the specific needs of different contexts and SES groups.

## Materials and Methods

### Design and Participants

We applied a cross-sectional design, with purposive sampling. Selected samples from different SES and contexts (rural/urban) were evaluated with cognitive tasks as well as individual and environmental variables. The minimum sample size required was calculated considering an anticipated effect size of 0.15, a statistical power level of 0.8, two predictors (context and UBN), and a probability level of 0.05 (Cohen et al., 2013). One hundred and thirty one 5-year-olds (68 males; mean age in years = 5.36, *SD* = 0.27) recruited in four public schools and their families participated in the study Table 1. Two of the schools were urban, located in the City of Buenos Aires, which has 2890151 habitants (Olinto et al., 2013, pp. 1–8) and is the city with the highest percentage of urban population in Argentina.

**Table 1:** Frequencies by socioeconomic level and context.

Number of UBN indicators	Rural	Urban
0	8	61
1	15	14
2	25	8
	48	83


The other two schools were located in Santiago del Estero, the province of that has the highest percentage of rural population in Argentina (31.3% of Santiago del Estero’s population is rural). Specifically, schools were in Añatuya’s outskirts, a city with 23286 habitants and an average density population of 6.3 habitants/km2. Based on Miller and Votruba-Drzal (2013, p. 234) ‘rural’ criteria (an area with population less than 50000 inhabitants which is independent of a metropolis -less than 30% of the population go to work to a metropolis-), both schools were considered rural.

All children attended the school on either the morning or afternoon shifts. The number of children on each shift did not differ by living context (*U* = 1685, *Z* = 1.736, *p* = 0.082). In addition, rural and urban children did not differ significantly by gender (χ^2^ = 1.636, df = 3, *p* = 0.651) or age [*F*_(1,126)_ = 0.214; *p* = 0.887]. All the schools provided children with a meal and a snack. Primary caregivers gave written informed consent to participate in the study, which was authorized by an institutional Ethical Committee (Centro de Educación Médica e Investigaciones Clínicas, Consejo Nacional de Investigaciones Científicas y Técnicas, Protocol N 967). The study was conducted in accordance with APA’s ethical standards, and international and national children rights laws.

### EF and Non-verbal Intelligence Assessment

#### Attention

To assess Attention, we used the Attention subtest of CUMANIN Battery (Children Neuropsychological Maturity Questionnaire) (Portellano et al., 2000, pp. 1–28), a cancelation task in which the child is given 30 s to identify and strike out the 20 geometric figures that are equal to a target (square) in a copy containing 100 figures.

#### Inhibitory Control

Day and Night like-Stroop test (Gerstadt et al., 1994, p. 129), in Tardif and collaborators variant (Tardif et al., 2007, p. 318), was applied to assess Inhibitory control. Children were presented a congruent block of 10 trials (where the child should say ‘day’ when seeing the sun card), followed by an incongruent block of 10 trials (where the child should say ‘night’ when seeing the picture of the sun).

#### Working Memory

Forward digit span subtest of WISC III (Wechsler Intelligence Scale for Children III) (Wechsler, 1991, p. 219) was used to evaluate Working memory. In this task, the child must repeat number sequences in the same order they were listened.

#### Flexibility

Dimensional Change Card Sort (DCCS) (Frye et al., 1995), in the standard version (Zelazo, 2006, p. 297) was used to assess Flexibility. This is a game of two dimensions cards that children should classify according to a first dimension (color) and then another dimension (shape). The outcome variable of this task was ordinal. Therefore, with this variable, we applied ordinal regressions.

#### Non-verbal Intelligence

The Test of Non-verbal Intelligence 4 (TONI-4) (Brown et al., 2010, p. 1) was applied to evaluate Non-verbal intelligence (Horn and Cattell, 1966, p. 253). Each item consists of a sequence of abstract figures in which the child must select the only option that complete the pattern.

#### Learning

The Subtest Coding of WPPSI III battery (Intelligence Scale Wechsler Preschool and Primary III) was implemented to assessed Learning ability (Wechsler, 2004, pp. 1–329). The child must copy a series of symbols within geometric shapes, during 2 min. Each symbol is paired with a geometric shape and the child has a model that reminds correspondences.

All tests were presented in the listed order. We considered as general Non-verbal intelligence measures only TONI-4 (Intelligence) and Coding (Learning) tasks. All the other tests were EF measures. The inclusion of EF and Non-verbal intelligence measures was aimed at obtaining a whole cognitive assessment, complementing data of general measures with data from basic executive processes tests.

### Evaluation of Individual and Environmental Variables

To obtain individual and environmental information of children, we performed interviews with parents or caregivers. Scales were administered in the following order:

#### Environmental Variables

The Socioeconomic Level Scale (NES) (Colombo and Lipina, 2005, pp. 1–173) was used to estimate: (1) the presence and number of indicators of Unsatisfied Basic Needs (UBN indicators) at home; (2) health history of the child; (3) home stimulation; and (4) demographic variables (e.g., age, gender, time of residence in the place).

#### Child Temperament

The very short form of the Child Behavioral Questionnaire (Putnam and Rothbart, 2006, p. 102) for children 3–7 years was administered to evaluate child temperament. The behavior of children is evaluated by their mothers following an 8-point scale according to how true is each behavior in the case of his son.

#### Mother Mental Health

The Hamilton Anxiety and Depression Scale (Hamilton, 1960, p. 56) which consists of 14 items related to signs and symptoms of anxiety and depression, and value the intensity and frequency of such behaviors during 20 days prior to the interview was used to obtain mother mental health indicators.

From all those three scales, we obtained 35 individual and environmental variables (list of variables in Tables 2, 3).

**TABLE 2:** Descriptive statistic of the continuous variables obtained from parents’ interviews in urban and rural contexts.

****	**Urban**			**Rural**		
**Continuous variables**	***n***	**Mean**	***SD***	***n***	**Mean**	***SD***
****Time of residence in the place	83	4.88	2.28	48	5.88	0.61
Number of siblings^1^	83	1.34	0.99	47	2.53	1.36
Birth order^2^	82	1.76	0.90	47	2.62	1.21
Health history	77	0.91	1.04	47	1.19	1.14
Pregnancy health history	77	0.21	0.41	45	0.27	0.45
Father’s age	73	35.93	7.38	43	32.60	7.23
Mother’s age	76	32.89	6.93	48	29.21	7.15
Number of dependents in the household^3^	82	3.74	1.40	47	5.02	1.66
Father’s completed level of education^4^	72	7.39	2.84	41	2.41	1.72
Mother’s completed level of education	79	7.22	3.30	43	2.93	2.31
Father’s occupation^5^	78	3.73	1.79	45	1.51	1.01
Mother’s occupation score	81	2.16	2.44	46	0.33	0.97
Number of government subsidies^6^	77	0.51	0.62	47	1.02	0.49
Dwelling score	77	11.26	1.47	47	8.62	2.13
Past preschool attendance^7^	77	21.97	9.62	47	7.91	5.75
Number of books at home	77	1.95	1.00	47	2.81	0.54
Frequency of mother–child play (by week)	76	5.14	2.40	47	4.62	2.85
Frequency of reading newspapers (by week)	67	2.49	2.28	46	3.83	2.69
Frequency of watching TV (by week)	73	5.66	2.14	47	4.83	3.04
Frequency of listening to the radio (by week)	71	1.92	2.83	47	4.30	3.34
Frequency of using computers (by week)	71	2.83	3.03	47	0.36	1.48
Frequency of using cellphone (by week)	66	1.74	2.61	47	2.62	3.19
Mother anxiety	67	8.91	3.54	37	9.08	3.93
Mother depression	67	5.46	4.10	37	5.62	4.04
Surgency	76	4.39	0.82	45	4.27	0.89
Negative affect	76	4.60	0.79	45	4.37	0.85
Effortful control	76	5.76	0.68	45	5.43	0.73
Age	83	5.35	0.28	43	5.38	0.26

*^1^Total number of siblings of the child.*

*^2^Order in which the child was born.*

*^3^Number of people that economically depends on the household.*

*^4^Code used by the INDEC (Instituto Nacional de Estadísticas y Censos, 2010) to measure educational level where 0 = no studied, 1 = primary school uncompleted; 3 = primary school completed; 6 = high school uncompleted; 9 = high school completed/college uncompleted; 10 = college completed/graduate school uncompleted; 12 = graduate school completed.*

*^5^Code used by the INDEC (Instituto Nacional de Estadísticas y Censos, 2010) to measure occupation in function of salary where 0 is unemployed; 1 is, e.g., peddler; 2 is. e.g., a street sweeper; 4 is, e.g., taxi driver; etc.*

*^6^Number of subsidies given by the government to that family.*

*^7^Amount of months that the child had attended school before the year of the study.*

**TABLE 3:** Descriptive statistic of the nominal variables obtained from parents’ interviews in urban and rural contexts.

****	**Urban**			**Rural**	
**Nominal variables**	**Values**	**Frequency**	**Valid percentage**	**Frequency**	**Valid percentage**
****Gender	Girls	37	44.6	26	54.2
	Boys	46	55.4	22	44.6
Parenting	Father and mother	62	74.7	40	83.3
	Mother	15	18.1	6	12.5
	Others	6	7.2	2	4.2
Low birth weight	No	66	91.7	32	88.9
	Yes	6	8.3	4	11.1
Preterm birth	No	68	75	36	81.8
	Yes	8	16.7	8	18.2
Potential central nervous system conditions	No	56	72.7	33	70.2
	Yes	21	27.3	14	29.8
Incubator	No	68	89.5	40	87
	Yes	8	10.5	6	13
Hospital internship	No	61	73.5	33	68.8
	Yes	16	19.3	15	31.3

### SES Assessment: Number of Unsatisfied Basic Needs Indicators

From the information obtained through the NES Scale we applied the UBN approach in order to have a chronic poverty measure. Following the national poverty criteria (Instituto Nacional de Estadísticas y Censos, 2010, p. 52) we considered an UBN home the one that has at least one of these indicators: (1) Subsistence capacity (UBN 1): head of household with incomplete primary school educational level, and more than four dependents. (2) School truancy (UBN 2): presence of school-aged children (6–15 years-old) who do not attend any educational system. (3) Inappropriate dwelling (UBN 3): the house is a hotel or pension, poor housing or other housing not built for residential purposes. (4) Sanitary deficiencies (UBN 4): home with no flush toilet. (5) Overcrowding (UBN 5): home in which the ratio of the total number of home members to the number of rooms used to sleep is equal or higher than 3. In a second step, we counted the number of UBN indicators in order to distinguish levels of SES. Within UBN homes, we can differentiate between those which have one (lower poverty level) and those that have two or more UBN indicators (higher poverty level).

### Evaluation of Context: Rural/Urban

Based on Miller and Votruba-Drzal (2013, p. 234) we considered as ‘rural’ an area with population less than 50000 inhabitants which is independent of a metropolis (less than 30% of the population go to work to a metropolis). All homes of children evaluated in the city of Añatuya meet the criteria to be considered rural. Similarly, we define urban as a city with a population higher than 150000. All homes of children evaluated in the Autonomous City of Buenos Aires meet that criterion.

### Statistical Analysis

Data is available in a repository (Hermida, 2018). Data from children with diagnosed developmental disabilities or neurological diseases were excluded from the study.

We conducted all analysis for each cognitive process separately in order to detect function-specific susceptibilities to environmental conditions at this time of development.

In the first step, to determine the contribution of UBN indicators (0, 1, or 2) and context (urban or rural) to performance on EF and intelligence tasks, we performed multiple linear regressions (Enter method) for continuous variables (attention, inhibitory control, working memory, intelligence, and learning) and an ordinal multiple regression for the ordinal variable (flexibility). We verified assumptions for linear (we conducted residual analyses to check normality, independence, homoscedasticity, and no collinearity) and for ordinal regressions (Pearson χ^2^ test to analyze goodness of fit and likelihood-ratio test of proportionality of odds to evaluate the proportional odds assumption). Context, UBN indicators, as well as the interaction between both, were included as independent variables.

In the second step, to determine which variables explained the effects on cognitive performance previously identified, we performed one two-way ANOVA for each of the 35 individual and environmental variables, including context and UBN indicators as factors. In the ANOVAs, to assure that differences were not generated by a non-homogenous UBN indicators distribution across both contexts, we added a non-parametric control of results through a permutation test (as was proposed by Maris and Oostenveld, 2007, p. 177). Furthermore, because of the high number of comparisons, we applied a Bonferroni correction to minimize potential error. For nominal variables (e.g., gender), we performed Kruskal Wallis tests. We selected the variables that only showed significant differences by context, i.e. variables that vary between rural and urban areas. We conducted correlations among those variables. In case of high correlation (*r* > 0.70), we selected one of them for the following analysis.

In the third step, to determine whether these selected variables were associated with EF and intelligence, we performed multiple linear/ordinal regressions (Enter method). In this model we included the selected variables (that differed between rural and urban contexts) as independent variables and one cognitive variable as the dependent variable. This model was applied to each of the six EF and intelligence variables.

## Results

Table 2 shows that, although the rural sample have, on average, higher scores on demographic characteristics linked to poverty (i.e., higher number of dependents in the household, lower father’s and mother’s completed level of education, lower father’s and mother’s occupation score, lower dwelling score and higher number of government subsides), some factors found in prior literature to be associated with children’s better cognitive functioning (Bradley et al., 2001, p. 1844), scored higher among the rural than the urban sample (i.e., number of books at home, frequency of reading newspapers, frequency of listening to the radio and frequency of using cellphones).

**FIGURE 1:**
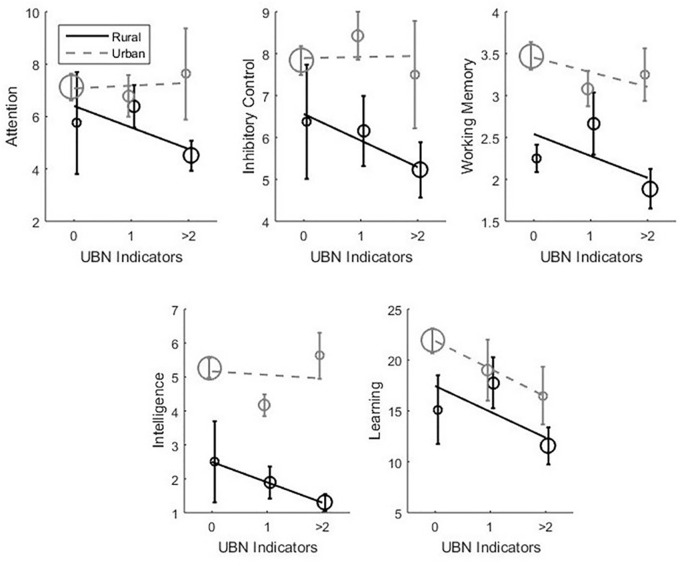
Cognitive achievement as a function of context (urban or rural) and SES (measured as the number of UBN indicators). Lines correspond to linear fits of each context separately; points’ sizes correspond to subsample sizes. Effects of UBN on flexibility are not shown in the figure because it is an ordinal variable.

The number of UBN indicators was larger in the rural (mean = 1.35, *SD* = 0.76) than in the urban sample (mean = 0.36, *SD* = 0.65) indicating that SES was lower in the rural context (descriptive statistics is in Tables 2–4; Pearson’s *r* = -0.57). However, critical to the objectives of this paper, context and SES showed enough within variability and overlapped across urban and rural groups, to allow disentangling their contribution in multiple linear regressions (see model fitting information in Table 5). We then calculated multiple linear regressions with UBN, context and the interaction as main factors (Figure 1 and Table 6), for all cognitive tasks except for Flexibility (ordinal variable). For Flexibility we calculated an ordinal regression, which showed an adequate goodness of fit [Pearson χ^2^(12) = 13.433; *p* = 0.338] and met the proportional odds assumption [χ^2^(4) = 6.63; *p* = 0.156]. Figure 1 reveals a general trend observed in the data across all cognitive measures: (1) cognitive performance decreases with UBN indicators and (2) for fixed values of UBN, performance is worse for the rural than for the urban context. Quantitative analyses of the multiple regressions showed that the effect of context accounted much more significantly and reliably for the variance of the data than the effect of UBN indicators. Context modulated Inhibitory Control, Working Memory and Intelligence. And also showed a marginally significant (*p* = 0.051) effect on learning. The effect of context was always in the same direction: urban scores are higher than rural scores (Table 5). Instead, while all the slopes (β) of cognitive function as a function of UBN numbers were negative, this effect was significant only for Flexibility and Learning. No significant effect of the interaction was found.

**TABLE 4:** Descriptive statistics of cognitive variables by context and UBN indicators.

****	**Context**					
****	**Rural**			**Urban**		
****	***n***	**Mean**	***SD***	***n***	**Mean**	***SD***
****Attention	41	5.34	3.60	79	7.11	3.88
Inhibitory control	43	5.72	3.34	83	7.90	2.69
Working memory	38	2.21	1.19	77	3.39	1.18
Intelligence	27	1.85	2.21	72	5.13	2.08
Learning	44	14.05	9.54	82	20.87	9.79
**Flexibility**	****	**Frequencies**	**Percentage**	****	**Frequencies**	**Percentage**
****No switch		19	43.18		14	17.50
0–3 switches		1	2.27		6	7.50
4–5 switches		8	18.18		5	6.25
6 switches		16	36.36		55	68.75

**Table 5:** Results of multiple linear regressions for each cognitive variable.

					Context		UBN indicators	
						
Dependent variables	*n*	df	F	*R*^2^	Standard β	*p*	Standard β	*p*
Attention	120	2, 119	3.10	0.050	0.187	0.080	-0.060	0.573
Inhibitory control	126	2, 125	8.08	0.116	0.299	0.004	-0.068	0.506
Working memory	115	2, 114	13.60	0.195	0.359	0.000	-0.133	0.181
Intelligence	99	2, 98	23.96	0.333	0.538	0.000	-0.085	0.349
Learning	126	2, 125	9.63	0.135	0.199	0.051	-0.218	0.032

			**χ^2^**	***R*^2^**	**Coefficient**	***p***	**Coefficient**	***p***

Flexibility	124	2, 123	22.86	0.083	0.075	0.454	-0.801	0.002


**TABLE 6:** Significant results of ANOVAs: environmental and individual variables that differ by context.

****	***F***	***p***	**Non parametric control *p*-value**	**η^2^**
****Number of siblings	20.151	0.000	0.000	0.178
Birth order	21.517	0.000	0.000	0.177
Number of dependents in the household	12.258	0.001	0.000	0.107
Father’s completed level of education	46.741	0.000	0.000	0.314
Father’s occupation	24.377	0.000	0.000	0.165
Number of government subsidies	13.334	0.000	0.000	0.099
Past preschool attendance	50.798	0.000	0.000	0.311

Once results suggested a major effect of context on cognitive performance, the following question emerged. Which individual and environmental aspects most prevalent in rural than in urban life posit greater risks for cognitive development? Our experimental study was well suited for this analysis, because we measured a wide distribution of 35 elements (including child health history, child temperament, home stimulation and sociodemographic variables, see variables in Tables 2, 3) that may be relevant to cognitive development in both populations.

To pursue this objective, we first identified which of the 35 variables differed by context and not by UBN. We performed two-way ANOVA for each of the 35 individual and environmental variables, including context and UBN indicators as factors. Variables that showed significant differences by context (but not by SES) were considered as candidates to inquire why rural context has such a stronger effect (for fixed UBN) compared to urban context. Significant results of ANOVAs, after Bonferroni correction, are informed in Table 6. Those analyses revealed that the following set of variables distinguished rural from urban populations: number of siblings, birth order (i.e., whether the child was first born, second born or so on), number of dependents in the household, father’s completed level of education, father’s occupation (i.e., whether the father is unemployed, construction worker, CEO, etc.), number of government subsidies and past preschool attendance (in months). As it is shown in Tables 2, 6, number of siblings, birth order, number of dependents in the household and number of government subsidies are significantly higher in the rural sample, while father’s completed level of education, father’s occupation and past preschool attendance are significantly higher in the urban sample.

Later, we conducted Pearson correlations between those variables and we found that number of siblings correlated with birth order (*r* = 0.83; *p* < 0.001). Therefore, we selected birth order for the following analysis.

**FIGURE 2:**
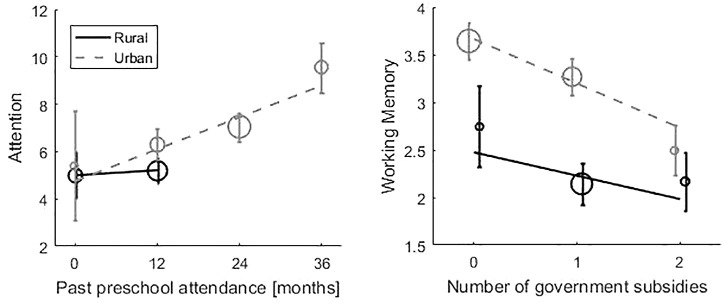
Cognitive achievement dependence with months of past preschool attendance and number of government subsidies. Lines correspond to linear fits of each context separately; points’ sizes correspond to subsample sizes. Rural scarceness is characterized by low past preschool attendance, high number of government subsidies and lower father’s completed level of education. Effects of Father’s completed level of education on flexibility are not shown in the figure because it is an ordinal variable.

Once this set of variables that differ between urban and rural contexts was identified we submitted it to multiple linear regressions to determine their predictive effect for each cognitive process. Regressions were conducted in rural and urban samples. Results showed that three of these variables had a significant effect on children cognitive performance (Figure 2): past preschool attendance, father’s completed level of education, and number of government subsidies. Months of past preschool attendance was positively correlated with Attention (*R*^2^ = 0.193, *F* = 3.678, β = 0.285, *p* = 0.026). Importantly, as Figure 2 shows, when months of past preschool attendance is matched, children in both contexts showed similar cognitive performance. Also, father’s completed level of education was correlated positively with Flexibility (*R*^2^ = 0.109, *Z* = 3.24, β = 0.265, *p* = 0.001). Finally, having more government subsidies was negatively associated with Working memory (*R*^2^ = 0.200, *F* = 3.670, β = -0.237, *p* = 0.046).

## Discussion

A large number of studies (Brooks-Gunn and Duncan, 1997, p. 55; Bradley and Corwyn, 2002, p. 371; Hackman and Farah, 2009, p. 65) have shown that growing in low SES conditions may present a significant risk for cognitive development. Here we show that within this general trend, for fixed levels of UBN, performance in a broad variety of cognitive measures, is worse for children living in rural compared to urban settings. Hence, following the model of risk accumulation proposed by Evans (2003, p. 924), to live in a poor place that also belongs to a rural area would imply even a greater risk.

This results are particularly relevant, since the incidence of extreme poverty is higher in rural areas and in children populations (Olinto et al., 2013, p. 125). Previous studies on children’s cognitive development in rural contexts have shown somehow non-linear results (Nadel and Sagawa, 2002, pp. 1–105; Wells and Evans, 2003, p. 311; Foulkes and Mori, 2009, p. 83; Kandawasvika et al., 2012, p. 1; Miller and Votruba-Drzal, 2013, p. 234; Miller et al., 2013, p. 234; Förster and Rojas-Barahona, 2014, p. 476; Mykerezi et al., 2014, p. 17; Tine, 2014, 599; Castro and Rolleston, 2015, pp. 5–48; Gouin et al., 2015, p. 1) and a meta analysis is very difficult to be performed because these studies are based on different cognitive measures, have used different age samples and, perhaps more importantly, have used different criteria to define rural contexts (Hart et al., 2005, p. 1149). In this controversial scenery, we provided a direct comparison between selected samples from different contexts, matched by age and for a wide spectrum of cognitive measures with overlapped distributions of UBN, albeit poverty was more pronounced in the rural sample. Although our sample size is small and each rural context has its own characteristics, it allowed us to factor out the distinct effects of context and poverty, and show that growing up in a rural setting (as the one of our sample) carries a higher risk for cognitive development.

Our study identified specifically two measures of scarceness that are more frequent in rural poverty and are decisive for cognitive function at a young age: lower past preschool attendance and lower father’s completed level of education. We discuss below the relevance of these measures. Here we stress an implication of this finding: rearing context does not impact cognition *per se*, but it does so through the lack of opportunities available in one context compared to the other. In fact, as it is shown in Figure 2, when months of past preschool attendance is matched, children in both contexts showed similar cognitive performance. Conversely, when cognitive performance was compared across contexts (Figure 1), for fixed number of UBN children in rural context showed worse performance. This indicates that attempting to collapse poverty in a single numerical indicator (as UBN) might be misleading, because different contexts pose distinct and singular risks to cognitive development (Lipina, 2016, p. 241; Duncan et al., 2017, p. 413).

In sum, the study shows that poverty in rural setting affects child cognitive performance more than urban poverty. These findings highlight the importance of using methodological designs that do not confound the effects of context and SES, though they might be correlated. Poverty in rural and urban contexts stands for qualitatively and quantifiable different forms of scarceness. These differences showed not only that rural poverty is more extreme than urban poverty, but also that it is more risky for child cognitive development. Importantly, this risk is not captured by typical poverty measures (SES, UBN) (Lipina et al., 2011, pp. 8–17; Lipina, 2016, p. 241; Duncan et al., 2017, p. 413). In turn, this questions the relevance and generalizability of interventions to promote child development based on studies performed in urban samples, which is the case for the majority of the research in the field (Schreuder, 2010, p. 45). Our results suggest that interventions could have different effects in rural and urban settings. Also, an intervention that works in one rural context may not work in another rural context. More studies are needed in rural contexts to have a big quantum of information available to design interventions specifically for rural children.

The fact that the months of past preschool attendance was found as one of the two decisive factors (within a broad list of 35 indicators) is quite revealing. The time spent in preschool had a very strong effect on attentional performance (ranging from 5 points to 10 points); an increment of 1 month in past preschool attendance is associated with an increase in target identification (i.e., 0.323 more targets identified) in 30-s task. At the same time, the graph shows that this form of scarceness is much more prominent in the rural environment (there were no children with more than 12 months of past preschool attendance in the rural sample). This finding resonates and is in line with a long tradition of investigation that has shown that the first years of education have an enormous impact on future cognitive development (Campbell et al., 2002, p. 42; Magnuson et al., 2004, p. 115; Temple and Reynolds, 2007, p. 126; Barnett, 2008, pp. 1–37; Pianta et al., 2009, p. 49; Heckman, 2011, p. 31; Brinkman et al., 2017, p. 483).

In this regard, various studies have documented the negative association between cost and preschool attendance. Results of studies with samples from Mexico City (Wong and Levine, 1992, pp. 89–102), Brazil (Connelly et al., 1996, pp. 619–656), Kenya (Lokshin et al., 2004, pp. 240–276), and Romania (Lokshin and Fong, 2006) suggest that financial constraints play key roles in families’ decisions about preschool attendance in developing countries. Conversely, our results showed that preschool attendance varied by context, but not by SES. A possible explanation for this result could be the lower number of preschool schools in rural contexts compared to urban ones (Gong et al., 2015, pp. 194–208). In the particular case of Argentina, evidence suggest that one of the main reasons of the urban-rural gap in preschool attendance is the lack of local preschool availability (Ferro, 2008, pp. 1–27) as it was also been reported in worldwide studies (Fuller et al., 2004, pp. 337–358; Bassok et al., 2011, pp. 7–19). However, due to other potential confounds (e.g., cultural factors) that we have not analyzed here, this hypothesis should be confirmed in future studies.

Our results encourage public policies oriented at maximizing access to preschool education in those contexts (Jiang et al., 2014, pp. 65–68), more so considering that in addition this may be protective from the impact of household deficits on cognitive development (Berry et al., 2016, p. 115).

The father’s completed level of education also explained why cognitive performance was lower for children in rural than that of children in urban settings. Regarding previous literature this result has both, a consistent and novel aspect. It is consistent with studies that associate parental education with cognitive development (Ardila et al., 2005, 539; Grantham-McGregor et al., 2007, p. 60; Bibok et al., 2009, p. 17). Rural parents in our sample have, in average, one less completed educational level than urban parents (Table 2), which is in line with previous studies showing lower parental education in rural settings, as well as lower educational opportunities (Nadel and Sagawa, 2002, pp. 1–105; Foulkes et al., 2008, p. 129; Foulkes and Mori, 2009, p. 83; Mykerezi et al., 2014, p. 17; Tine, 2017, pp. 9–22). Labor at early age (which has been shown to be a strong predictor of school drop-out), as well as distance and major costs of going to school, could explain why father’s level of education in rural contexts, is lower than in urban settings (Bedi and Marshall, 1999, pp. 657–682). In turn, low educational level is associated with early need of dropping out due to labor and with retention (Psacharopoulos, 1997, pp. 377–386; Bridgeland et al., 2006, pp. 1–44; Behrman et al., 2017, pp. 657–697). It is likely that this circle of scarceness, is more frequent in rural than in urban low SES contexts.

However, most studies in child development emphasized maternal over paternal education (Walker et al., 2007, p. 145). Little research is found regarding fathering and EF. Our result is novel because father’s education explains better rural and urban differences on child development than that of the mother’s (mother’s completed educational level varied according context and SES). Therefore, although father education has not been studied extensively, our research suggests that this variable is a candidate to explain the rural-urban gap in EF.

The third variable to explain why cognitive performance was worse for children in rural than urban settings, was number of government subsidies. The pathways though which subsidies impact child cognitive development are still unclear, and might be multiple and non-linear. For instance, the type of subsidy (Baird et al., 2014, pp. 1–43), amount of cash (Bourguignon et al., 2003, pp. 229–254; Havnes and Mogstad, 2011, pp. 97–129), and recipient of the subsidy (Das, 2005, pp. 57–80) can influence that association. Besides this complex scenario, it is well established that government subsidies contribute to better cognitive achievement worldwide (Heckman, 2006, p. 1900) and this result has been demonstrated specifically in the case of Argentina (Roca, 2008, pp. 315–330; Agis et al., 2013, pp. 1–77). Therefore, the fact that number of subsidies was negatively associated with working memory, may seem paradoxical. However, it can be understood since government subsidies are a proxy measure of the precariousness of living conditions (it is given only to families with unemployed parents living in vulnerable living conditions). Hence, homes that receive more government subsidies are also homes that have the most vulnerable living conditions (Roca, 2011, pp. 30–43), in our study, the rural sample. This indicates that while government subsidies are effective (Roca, 2008, pp. 315–330; Roca, 2011, pp. 30–43; Agis et al., 2013, 1–77), their effect measured in variability in cognitive performance is insufficient to compensate —in Argentina– the original differences in income and social resources between urban and rural settings. Future studies should analyze under which conditions subsidies are more effective in urban or rural contexts and which are the specific characteristics a government subsidy has to have in order to bridge the urban-rural gap in child cognitive achievement.

In summary, the factorial study of dimensions of poverty we performed confirms that living in a rural area does not limit cognitive opportunities *per se*. Instead, certain forms of low SES that are typical of rural areas have a strong impact in cognitive development. This might explain the contradictory results of previous studies on the impact of living context on cognition (Wells and Evans, 2003, p. 311; Grace et al., 2006, pp. 1–28; McGrail and Humphreys, 2009, p. 124; Kandawasvika et al., 2012, p. 1; Förster and Rojas-Barahona, 2014, p. 476; Mykerezi et al., 2014, p. 17; Tine, 2014, p. 599; Castro and Rolleston, 2015, pp. 5–48; Gouin et al., 2015, p. 1). Here we measured the impact of living context on cognition trough standardized non-verbal intelligence scores as well as EF scores. Considering that EF are strong predictors of future cognitive development and well-being (McClelland, 1973, p. 1; Brooks-Gunn and Duncan, 1997, p. 55; Blair and Diamond, 2008, p. 899), our findings suggest that this scenario of lower achievements for children living in a low SES rural context is likely to condition their future lives, if no intervention mediates that path. Moreover, the effect of government subsidies—which in Argentina over the last few years have improved the quality of life of vulnerable families (Roca, 2008, pp. 315–330; Roca, 2011, pp. 30–43; Agis et al., 2013, pp. 1–77)—is insufficient to overcome these risks.

While more studies are required, our findings and many others (Slavin, 2002, p. 15; Yoshikawa et al., 2012, p. 272; Richter et al., 2017, p. 103) suggest that investment in early education may be a shield for a healthy cognitive development that stretches into adulthood. Regarding this issue, our results (Table 2) highlight some factors present in rural context, that, given the constraints in rural settings, might be facilitators for interventions. For example, the higher frequency of using cellphones, listening to the radio and reading newspapers, might be used to the transmission of early development tips to mothers, or to the presentation of cognitive training activities directly to children. Some interventions have showed promising results with similar methods (Gruver et al., 2016, p. 159; Nieuwboer et al., 2017, p. 61).

It is important to emphasize that while here we have studied typical urban and rural contexts in South America, the social and demographic characteristics of these contexts may vary in different regions of the planet. Also, our study is based on a middle size sample and has the limitation of not having rural-urban continuum, so we cannot draw conclusions regarding the intermediate contexts such as suburbs or small towns (Foulkes and Mori, 2009, p. 83). Hence, the conclusions of this study have to be understood relative to this particular region of the world. Nevertheless, our study raises a general concern about studies of low SES and cognition by signaling that poverty scores may provide insufficient and erroneous characterizations of a population. Instead, understanding what dimensions of scarceness characterize a population might open a wider window for our understanding of the adequacy of educational and governmental interventions that target the prevention of deficits in cognitive development.

## Conclusion

As poverty is more extreme in rural settings, the effects of context (rural/urban) and socioeconomic status (SES) are often confounded. In this paper we isolated these effects and showed that living in a non-urban context has a negative impact on children’s cognitive performance that is independent of SES and more pronounced than that of low SES. Poverty in rural and urban contexts imply qualitatively and quantifiable different forms of scarceness. Factors including fewer months of past preschool attendance as well as a lower completed level of education of fathers, typical of rural contexts, explained that, for the same level of SES, children in rural settings performed consistently worse than children in urban settings. These results have implications for the design of public policy and intervention programs that aim to address the needs of specific living contexts and socioeconomic groups.

## Data Availability Statement

The datasets generated and analyzed for this study can be found in the PsyArxiv https://mfr.osf.io/render?url=https://osf.io/65z2y/?action=download%26mode=render.

## Ethics Statement

This study was carried out in accordance with the recommendations of APA’s ethical standards. All subjects gave written informed consent in accordance with the Declaration of Helsinki. The protocol was approved by the Ethical Committee of the Centro de Educación Médica e Investigaciones Clínicas “Norberto Quirno”.

## Author Contributions

MH, MSS, AG, SL, and MS designed the experiments. MH collected the data. MA contributed in the logistic organization for the fieldwork in the rural setting. MH, DS, MSS, SL, and MS worked in the data analysis and interpretation. MH and MS wrote the manuscript. All co-authors revised and contributed to manuscript revision, read and approved the submitted version.

## Conflict of Interest Statement

The authors declare that the research was conducted in the absence of any commercial or financial relationships that could be construed as a potential conflict of interest.
