# Corrigendum: Risks for Child Cognitive Development in Rural Contexts

**DOI:** 10.3389/fpsyg.2021.647039

**Published:** 2021-02-11

**Authors:** Maria Julia Hermida, Diego Edgar Shalom, María Soledad Segretin, Andrea Paula Goldin, Marcelo Claudio Abril, Sebastián Javier Lipina, Mariano Sigman

**Affiliations:** ^1^Laboratorio de Neurociencia, Universidad Torcuato Di Tella, Buenos Aires, Argentina; ^2^Fundación Mundo Sano, Buenos Aires, Argentina; ^3^Instituto de Educación, Universidad Nacional de Hurlingham, Buenos Aires, Argentina; ^4^Consejo Nacional de Investigaciones Científicas y Técnicas, Buenos Aires, Argentina; ^5^Departamento de Física, Facultad de Ciencias Exactas y Naturales, Universidad de Buenos Aires, Buenos Aires, Argentina; ^6^Unidad de Neurobiología Aplicada, Centro de Educación Médica e Investigaciones Clínicas “Norberto Quirno”, Buenos Aires, Argentina; ^7^Facultad de Lenguas y Educación, Universidad Nebrija, Madrid, Spain

**Keywords:** socioeconomic status, rural context, urban context, child cognitive development, executive functions, preschool attendance, father's educational level

In the original article, there were mistakes in **Tables 2**, **3**, **4**, and **6** as published.

There were three mistakes in **Table 2**. Firstly, column “Media” should be column “Mean.” Secondly, in reference 6 (footnote) “subsides” should be “subsidies.” Thirdly, in row “Frequency of mother child play (by week),” column “Urban/Mean” it says 2.14 and it should say 5.14.

There were two mistakes in **Table 3**. In row “Incubator/yes,” column “Urban/valid percentage,” it says 1.5 and it should say 10.5. Also, in row “Potential Nervous System conditions: No,” column “Rural/ Valid percentage,” it says 7.2 and it should say 70.2.

There were four mistakes in **Table 4**. Firstly, in rows “0–3 switchs,” “4–5 switchs,” and “6 switchs,” the word “switchs” should be “switches.” Additionally, in row “Learning,” column “Urban/mean,” it says 2.87 and it should say 20.87.

Finally, there were two mistakes in **Table 6**. In row “Number of siblings,” column “F,” it says 2.151 and it should say 20.151. Also, in row “Past preschool attendance,” column “F,” it says 5.798 and it should say 50.798.

The corrected Tables 2, 3, 4, and 6 appear below.

**Table 2:** Descriptive statistic of the continuous variables obtained from parents' interviews in urban and rural contexts.

	**Urban**			**Rural**		
**Continuous variables**	***n***	**Mean**	***SD***	***n***	**Mean**	***SD***
Time of residence in the place	83	4.88	2.28	48	5.88	0.61
Number of siblings^1^	83	1.34	0.99	47	2.53	1.36
Birth order^2^	82	1.76	0.90	47	2.62	1.21
Health history	77	0.91	1.04	47	1.19	1.14
Pregnancy health history	77	0.21	0.41	45	0.27	0.45
Father's age	73	35.93	7.38	43	32.60	7.23
Mother's age	76	32.89	6.93	48	29.21	7.15
Number of dependents in the household^3^	82	3.74	1.40	47	5.02	1.66
Father's completed level of education^4^	72	7.39	2.84	41	2.41	1.72
Mother's completed level of education	79	7.22	3.30	43	2.93	2.31
Father's occupation^5^	78	3.73	1.79	45	1.51	1.01
Mother's occupation score	81	2.16	2.44	46	0.33	0.97
Number of government subsidies^6^	77	0.51	0.62	47	1.02	0.49
Dwelling score	77	11.26	1.47	47	8.62	2.13
Past preschool attendance^7^	77	21.97	9.62	47	7.91	5.75
Number of books at home	77	1.95	1.00	47	2.81	0.54
Frequency of mother-child play (by week)	76	5.14	2.40	47	4.62	2.85
Frequency of reading newspapers (by week)	67	2.49	2.28	46	3.83	2.69
Frequency of watching TV (by week)	73	5.66	2.14	47	4.83	3.04
Frequency of listening to the radio (by week)	71	1.92	2.83	47	4.30	3.34
Frequency of using computers (by week)	71	2.83	3.03	47	0.36	1.48
Frequency of using cellphone (by week)	66	1.74	2.61	47	2.62	3.19
Mother anxiety	67	8.91	3.54	37	9.08	3.93
Mother depression	67	5.46	4.10	37	5.62	4.04
Surgency	76	4.39	0.82	45	4.27	0.89
Negative affect	76	4.60	0.79	45	4.37	0.85
Effortful control	76	5.76	0.68	45	5.43	0.73
Age	83	5.35	0.28	43	5.38	0.26

1 Total number of siblings of the child.

2 Order in which the child was born.

3 Number of people that economically depends on the household.

4 Code used by the INDEC (Instituto Nacional de Estadísticas y Censos, 2010) to measure educational level where 0 = no studied, 1 = primary school uncompleted; 3 = primary school completed; 6 = high school uncompleted; 9 = high school completed/college uncompleted; 10 = college completed/graduate school uncompleted; 12 = graduate school completed.

5 Code used by the INDEC (Instituto Nacional de Estadísticas y Censos, 2010) to measure occupation in function of salary where 0 is unemployed; 1 is, e.g., peddler; 2 is. e.g., a street sweeper; 4 is, e.g., taxi driver; etc.

6 Number of subsidies given by the government to that family.

7 *Amount of months that the child had attended school before the year of the study*.

**Table 3:** Descriptive statistic of the nominal variables obtained from parents' interviews in urban and rural contexts.

	**Urban**			**Rural**	
**Nominal variables**	**Values**	**Frequency**	**Valid percentage**	**Frequency**	**Valid percentage**
Gender	Girls	37	44.6	26	54.2
	Boys	46	55.4	22	44.6
Parenting	Father and mother	62	74.7	40	83.3
	Mother	15	18.1	6	12.5
	Others	6	7.2	2	4.2
Low birth weight	No	66	91.7	32	88.9
	Yes	6	8.3	4	11.1
Preterm birth	No	68	75	36	81.8
	Yes	8	16.7	8	18.2
Potential central nervous system conditions	No	56	72.7	33	70.2
	Yes	21	27.3	14	29.8
Incubator	No	68	89.5	40	87
	Yes	8	10.5	6	13
Hospital internship	No	61	73.5	33	68.8
	Yes	16	19.3	15	31.3

**Table 4:** Descriptive statistics of cognitive variables by context and UBN indicators.

	**Context**					
	**Rural**			**Urban**		
	***n***	**Mean**	***SD***	***n***	**Mean**	***SD***
Attention	41	5.34	3.60	79	7.11	3.88
Inhibitory control	43	5.72	3.34	83	7.90	2.69
Working memory	38	2.21	1.19	77	3.39	1.18
Intelligence	27	1.85	2.21	72	5.13	2.08
Learning	44	14.05	9.54	82	20.87	9.79
**Flexibility**		**Frequencies**	**Percentage**		**Frequencies**	**Percentage**
No switch		19	43.18		14	17.50
0–3 switches		1	2.27		6	7.50
4–5 switches		8	18.18		5	6.25
6 switches		16	36.36		55	68.75

**Table 6:** Significant results of ANOVAs: environmental and individual variables that differ by context.

	***F***	***p***	**Non parametric control *p-*value**	**η^2^**
Number of siblings	20.151	0.000	0.000	0.178
Birth order	21.517	0.000	0.000	0.177
Number of dependents in the household	12.258	0.001	0.000	0.107
Father's completed level of education	46.741	0.000	0.000	0.314
Father's occupation	24.377	0.000	0.000	0.165
Number of government subsidies	13.334	0.000	0.000	0.099
Past preschool attendance	50.798	0.000	0.000	0.311

The authors apologize for these errors and state that they do not change the scientific conclusions of the article in any way. The original article has been updated.
